# Conceptualisation of Empathy in Interactions Between Healthcare Professionals and People With Fibromyalgia Syndrome: A Mixed-Methods Study

**DOI:** 10.1007/s10880-025-10117-w

**Published:** 2025-12-29

**Authors:** Maria Planes Alias, David J Moore, Nicholas Fallon, Katie Herron, Charlotte Krahé

**Affiliations:** 1 School of Psychology, Liverpool John Moores University, Liverpool, United Kingdom; 2 Department of Psychology, Institute of Population Health, University of Liverpool, Liverpool, United Kingdom; 3 Pain Management Programme, The Walton Centre NHS Foundation Trust, Liverpool, United Kingdom

**Keywords:** Fibromyalgia Syndrome, Chronic pain, Clinical empathy, Patient- HCP communication, Q-sort Study

## Abstract

Psychological and relational processes, including empathy, are increasingly recognised as central to effective pain care. Fibromyalgia Syndrome (FMS), a complex chronic pain condition, poses significant challenges for both patients and healthcare professionals (HCPs) across medical settings. Patients with FMS often report lack of empathy from HCPs, negatively affecting healthcare experiences. Whilst empathic therapeutic relationships are linked to improved satisfaction and reduced pain in FMS, how empathy is conceptualised in practice remains underexplored. Using Q-methodology, 20 HCPs and 20 patients with FMS ranked 40 statements on clinical empathy based on agreement/disagreement. Four factors were identified, explaining 51% of the variance. Factor 1, ‘*Empathy is about truly connecting—the dominant healthcare professional view’*, included 75% of the HCPs and emphasised emotional aspects and partnership. The remaining factors captured heterogeneous patient perspectives: Factor 2, *‘Empathy cannot be taught; it is something that you have’*, focussed on behavioural, outcome-oriented aspects; Factor 3, *‘Empathy requires communication that goes both ways’,* prioritised behavioural and cognitive aspects; and Factor 4, *‘Lack of empathy makes patients feel abandoned—the dominant patient view’,* reflected a strong importance of emotional validation and personalised care. These four factors were further mapped onto broader dimensions of affective engagement and reciprocity. HCPs and patients view empathy differently, highlighting the need for interventions grounded in shared understanding. Addressing these perspectives may facilitate better empathic interactions and improve psychologically informed healthcare for FMS.

**Supplementary Information:**

The online version contains supplementary material available at 10.1007/s10880-025-10117-w.

## Introduction

Psychological and relational processes are increasingly recognised as central to effective pain care (Driscoll et al., 2021; Lakke & Meerman, 2016). Patients’ experiences of chronic pain are strongly shaped by interpersonal dynamics, such as patient-clinician communication (Armentor, 2017). Fibromyalgia Syndrome (FMS) is a prevalent and debilitating condition characterised by persistent widespread musculoskeletal pain and tenderness, physical and mental fatigue, cognitive disturbances, and psychological distress (Bhargava & Goldin, 2025). These symptoms profoundly impair functioning and quality of life (Al Sharie et al., 2024; Ashe et al., 2017), making FMS a paradigmatic condition for advancing psychologically informed pain care (Adams et al., 2023). FMS presents significant clinical challenges worldwide (Filipovic et al., 2025), which in the UK are further compounded by inconsistent services, limited care pathways, and unmet needs within the National Health Service (NHS; Wilson et al., 2022). Additionally, the absence of visible symptoms and definitive diagnostic biomarkers has led FMS to be labelled an “invisible” illness (Malluru et al., 2025), undermining its legitimacy and contributing to stigma (Mengshoel et al., 2018). This invisibility can affect how healthcare professionals (HCPs) express empathy (Paul-Savoie et al., 2018), potentially exacerbating both psychological and physical symptoms experienced by patients with FMS (Hickling et al., 2024).

A key psychosocial challenge for patients with FMS is the perceived lack of empathy from others, especially from HCPs (Colombo et al., 2025). Clinical empathy is commonly conceptualised as a multifaceted construct comprising four dimensions (Morse et al., 1992): affective (sharing another’s feelings), cognitive (understanding another’s perspective), behavioural (communicating that understanding), and moral (an altruistic motivation to be empathic), although the latter has not been consistently retained in later models (Decety & Jackson, 2004). This framework continues to be widely recognised in contemporary healthcare research and practice (Hojat, 2016; Jeffrey, 2016), with these dimensions collectively shaping interpersonal dynamics, including patient–provider communication (Mercer & Reynolds, 2002; Weisz & Cikara, 2021). Empathy represents a crucial yet relatively underexplored aspect of psychological pain care, particularly in “invisible” chronic conditions like FMS, where interpersonal factors strongly influence the course and management of the condition (Campos et al., 2024).

Many patients with FMS report feeling invalidated during clinical encounters (Hasselroth et al., 2021; Nishikawara et al., 2023), which is associated with poorer health outcomes, including increased pain perception and reduced quality of life (Lobo et al., 2014). Invalidation involves rejecting or dismissing a patient’s pain experience, often when no clear physical cause is found, whilst validation—the recognition and legitimisation of that experience—can be seen as a behavioural manifestation of empathy (Cano et al., 2008; Nicola et al., 2021, 2022). In the context of FMS, where patients face the stigma of “invisible” illness, validation may play a critical role in shaping patient-provider relationships and therapeutic outcomes. The current study, however, focussed on empathy as a broader construct encompassing affective, cognitive, and behavioural dimensions, with validation representing one key expression.

Despite its recognised importance, how empathy is communicated and perceived in medical settings remains insufficiently understood. Although research increasingly investigates the experiences and challenges of both patients with FMS and HCPs (Briones-Vozmediano et al., 2013; Byrne et al., 2023; Hayes et al., 2010), their underlying conceptualisations of clinical empathy have yet to be examined. Clarifying these conceptualisations is essential to determine whether or not patients and HCPs share a common understanding of empathy, and how this knowledge can inform improvements in healthcare interactions.

Accordingly, the present study employed Q-methodology to compare the perspectives of HCPs and individuals with FMS on clinical empathy. This mixed-methods approach combines quantitative rigour with qualitative depth (Kamal et al., 2014), enabling systematic study of complex, subjective constructs like empathy (Akhtar-Danesh et al., 2008). Its application is growing in healthcare and pain research, where it effectively captures perspectives shaped by social interactions and lived experiences (Churruca et al., 2021; McParland et al., 2011; Parsons et al., 2024). Whilst Q-methodology has been applied separately to explore perspectives from either patients with FMS (Kool et al., 2009) or HCPs (Scott et al., 2023), no research has examined both groups concurrently for direct comparison. Our objective was to explore how empathy is understood by HCPs and patients with FMS, thereby aiming to contribute to more empathic interactions in FMS care.

## Methodology

### Design

This pre-registered study (https://osf.io/pkvu7) followed a standard two-phase Q-methodology design (Watts & Stenner, 2012). In Phase 1, a “Q-set” of 40 statements on clinical empathy was co-developed with individuals living with chronic pain, including FMS, and specialists in pain management. Statement development was guided by established principles of Q-methodology (Paige & Morin, 2016; Webler et al., 2009) to ensure clarity, neutrality, and conceptual diversity to capture the complexity of empathy in clinical interactions (see *The Q-Set* below).

In Phase 2, a separate group of people with FMS (*n* = 20) and HCPs (*n* = 20) were recruited to complete the Q-Sort study. In the sorting task, participants ranked the statements of the Q-set based on their level of agreement/disagreement with each statement, producing individual “Q-sorts” that reflected their viewpoints on clinical empathy. After the task, participants provided qualitative reflections on the sorting process and on the statements placed at the extremes. To identify viewpoints, we applied by-person factor analysis, which assigns individuals to factors based on their Q-sort rankings and reveals distinct viewpoints, further enriched by participants’ qualitative comments (see *Plan of Analysis*).

### Participants

Ethical approval was granted in November 2024 by the Health Research Authority and Health and Care Research Wales, United Kingdom (IRAS Project ID: 342770).

A formal power analysis or statistical sample size calculation was not required as Q-methodology focuses on identifying and describing distinct viewpoints rather than making population-level inferences (McHugh et al., 2019; Watts & Stenner, 2012). Instead, practical recommendations suggest recruiting 30–40 participants to ensure a broad range of perspectives (Akhtar-Danesh et al., 2008). Our final sample of 40 participants—20 individuals with FMS and 20 HCPs—therefore aligns with these recommendations. With two participant groups, the target of 20 per group also adheres to the guideline of recruiting about half the number of participants as there are statements—40 in this Q-set—to achieve a stable factor structure (Watts & Stenner, 2012).

Participants were eligible to take part if they were aged 18 years or older, proficient in English, and residing in the United Kingdom. Patients required a formal FMS diagnosis and must have sought clinical help for FMS within the past year. Patients employed in healthcare roles involving interactions with people with FMS were excluded. HCPs had to be working in a UK healthcare service and have seen at least one patient with FMS in the past 12 months; retired HCPs and HCPs seeking help for their own FMS or chronic pain were excluded. Individuals who contributed to Q-set generation were also ineligible to take part.

All participants completed the Q-sort online and were recruited through purposive sampling via email invitations. Patients with FMS were identified from the Pain Management Registry at The Walton Centre NHS Foundation Trust and contacted only by its research lead. This registry includes individuals diagnosed with chronic pain who have consented to be contacted for research (REC reference: 24/NW/0068). HCPs were identified and contacted through the research team’s professional networks. All participants provided informed consent prior to participation and completed a screening questionnaire to confirm eligibility.

The final sample of HCPs were employed in NHS trusts or equivalent public health services, with only one working across both public and private sectors. As summarised in Table 1, they varied in professional experience, care levels, fields of practice, and pain expertise, with most (*n* = 12) holding advanced/consultant-level expertise. Participants with FMS reported a range of pain durations and levels of perceived support from clinical and non-clinical sources.

**Table 1:** Descriptive statistics for demographic and sample characteristics

HCP Group (*N* = 20)					
**Variables**		**Factor 1** (*n =* 15) N (%)	**Factor 2** (*n =* 1) N (%)	**Factor 3** (*n =* 1) N (%)	**Factor 4** (*n =* 3) N (%)
Gender	Female	9 (60%)	–	1 (100%)	3 (100%)
	Male	6 (40%)	1 (100%)	-	-
Self-identified as neuro-divergent	No	14 (93.3%)	1 (100%)	1 (100%)	2 (66.7%)
	Yes	1 (6.7%)	–	–	1 (33.3%)
Job duration^a^	< 3 years	3 (20%)	–	–	2 (66.7%)
	3–5 years	5 (33.3%)	1 (100%)	–	-
	5–10 years	1 (6.7%)	–	–	-
	> 10 years	6 (40%)	–	1 (100%)	1 (33.3%)
Main field of work^b^	Medicine	3 (20%)	–	-	-
	Allied healthcare	5 (33.3%)	–	1 (100%)	1 (33.3%)
	Mental health	7 (46.7%)	1 (100%)	–	2 (66.7%)
Level of care^c^	1ry	3 (20%)	–	–	-
	1ry and 2ry	1 (6.7%)	–	–	-
	2ry	7 (46.7%)	1 (100%)	–	1 (33.3%)
	2ry and 3ry	2 (13.3%)	–	–	-
	3ry	2 (13.3%)	–	1 (100%)	2 (66.7%)
Pain expertise^d^	Limited—Moderate	3 (20%)	1 (100%)		1 (33.3%)
	Experienced but non-specialist	2 (13.3%)	–	–	1 (33.3%)
	Advanced expertise / Pain specialist	10 (66.7%)	–	1 (100%)	1 (33.3%)

FMS Group (*N =* 20)					
Variables		**Factor 1** (*n =* 3) N (%)	**Factor 2** (*n =* 3) N (%)	**Factor 3** (*n =* 5) N (%)	**Factor 4** (*n =* 9) N (%)
Gender^e^	Female	2 (66.7%)	2 (66.7%)	3 (60%)	8 (88.9%)
	Male	1 (33.3%)	1 (33.3%)	1 (20%)	1 (11.1%)
Self- identified as neuro-divergent	No	3 (100%)	1 (33.3%)	1 (20%)	5 (55.6%)
	Yes	–	2 (66.7%)	4 (80%)	4 (44.4%)
Pain duration	1–3 years	–	–	–	2 (22.2%)
	3–5 years	2 (66.7%)	2 (66.7%)	1 (20%)	4 (44.4%)
	5–10 years	1 (33.3%)	1 (33.3%)	2 (40%)	-
	> 10 years	–	–	2 (40%)	3 (33.3%)
		M (SD)	M (SD)	M (SD)	M (SD)
Self-reported perceived support^f^	Clinical	72.3 (14.3)	63 (28.6)	50 (28.9)	40 (43.6)
	Non-clinical	68.3 (28.4)	86 (3.61)	42.2 (28.9)	68.7 (19.6)

NOTE*. M* = mean; *SD* = standard deviation; HCP = Healthcare Professional; FMS = Fibromyalgia Syndrome. All demographic, screening, and exploratory variables were complete for all participants (*N =* 40). Percentages are relative to the total number of responses for each variable.

^**a**^Job duration indicates the number of years a professional has worked in healthcare.

^**b**^For ‘Main field of work,’ only categories represented in the sample are shown; other available options included nursing, pharmacy, public health, research/academia, and others.

^**c**^‘Level of care’ indicates the healthcare system layer: primary (1ry; first contact, e.g., GPs), secondary (2ry; specialist services, e.g., rheumatologists), and tertiary (3ry; advanced care, e.g., specialist pain clinics).

^**d‘**^Pain expertise’ asked about HCPs’ level of specialisation in chronic pain management.

^**e**^One participant in the FMS group selected "Prefer not to say" for gender, resulting in *n =* 4 for Factor 3.

^**f**^Perceived support from clinical and non-clinical sources was assessed using a scale from 0 (very poor support networks) to 100 (very strong support networks).

### Materials and Measures

#### The Q-Set

**Fig. 1:**
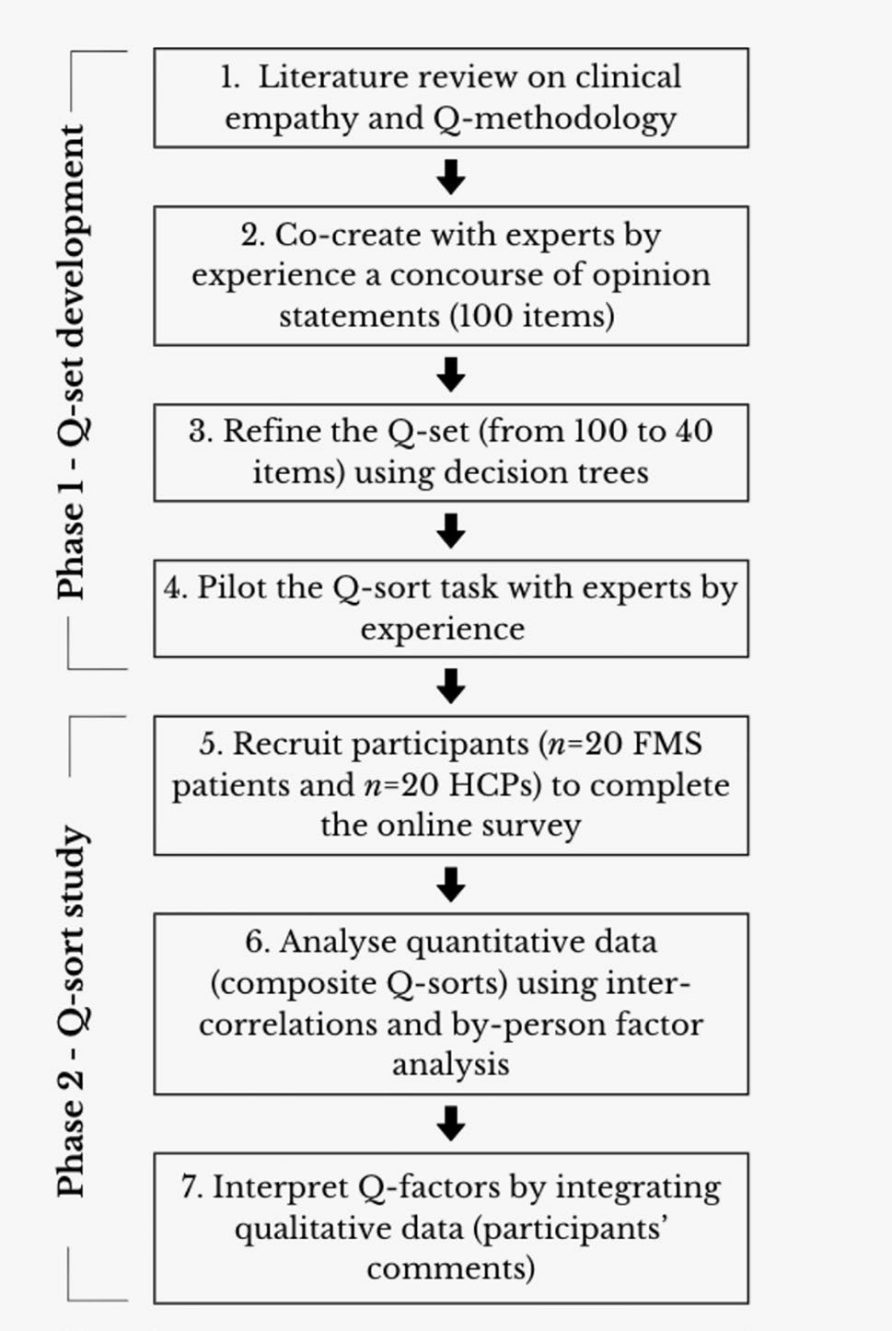
Steps in Q-set development and sorting task. NOTE: Experts by experience refer to people living with chronic pain and working in pain management

A structured Q-set was developed through consultations with four individuals living with chronic pain (including FMS) and two HCPs specialising in pain management: a clinical psychologist and a physiotherapist. These contributors participated in three sessions: one to brainstorm and generate initial statements, another to refine the item pool, and a final session to review the Q-set and pilot the sorting task. Figure 1 illustrates the steps followed in the Q-set development and Q-sort task (see *Supplementary Materials* for a detailed description of each phase).

Whilst the number of items in a Q-set can vary widely—from fewer than 20 to over 250 statements—Q-methodology typically employs around 40 statements (Churruca et al., 2021). Accordingly, the initial concourse of 100 statements was systematically refined to a 40-item Q-set using a structured process guided by two decision trees adapted from a recent Q-sort study (Bell et al., 2025). One tree evaluated individual items, whilst the other assessed the overall Q-set structure, ensuring transparency and replicability (see Figures S1 and S2). Statements were classified according to the affective, cognitive, and behavioural dimensions of clinical empathy (Morse et al., 1992), without requiring equal distribution across categories. The final 40-item Q-set is presented within the composite Q-sort grids by factor (see *Results* section).

#### The Q-Sort

**Fig. 2:**
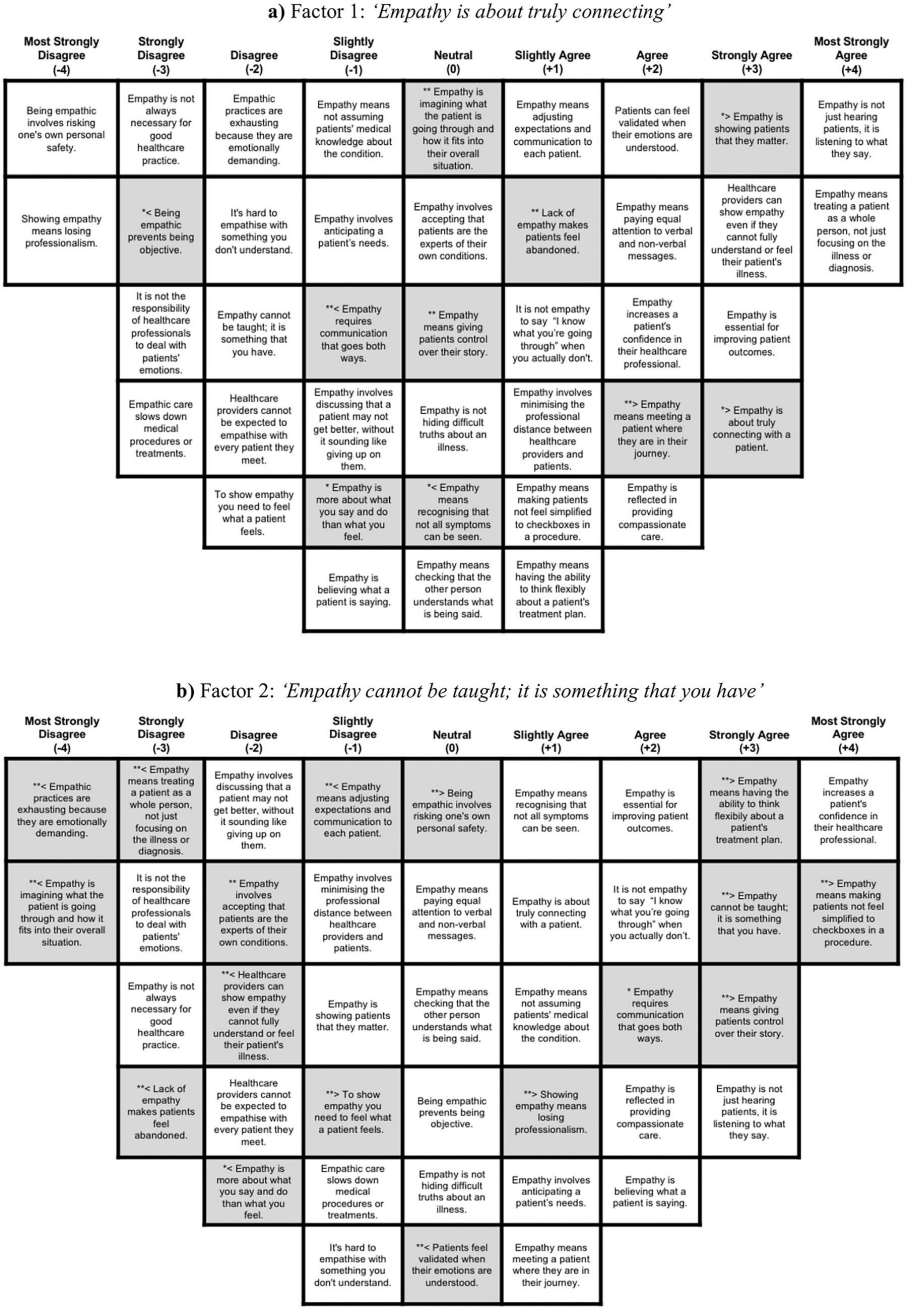
Composite Q-sorts for Factors 1 (panel a) and 2 (panel b). NOTE: Distinguishing statements are highlighted in grey. * = statistically distinguishing statement at .05 level; ** = statistically distinguishing statement at .01 level; ≥ z score indicates higher loading on this factor compared with other factors; ≤ z score indicates lower loading on this factor compared with other factors. Colour-coded versions of these grids are provided in Supplementary Figures S4 and S5

**Fig. 3:**
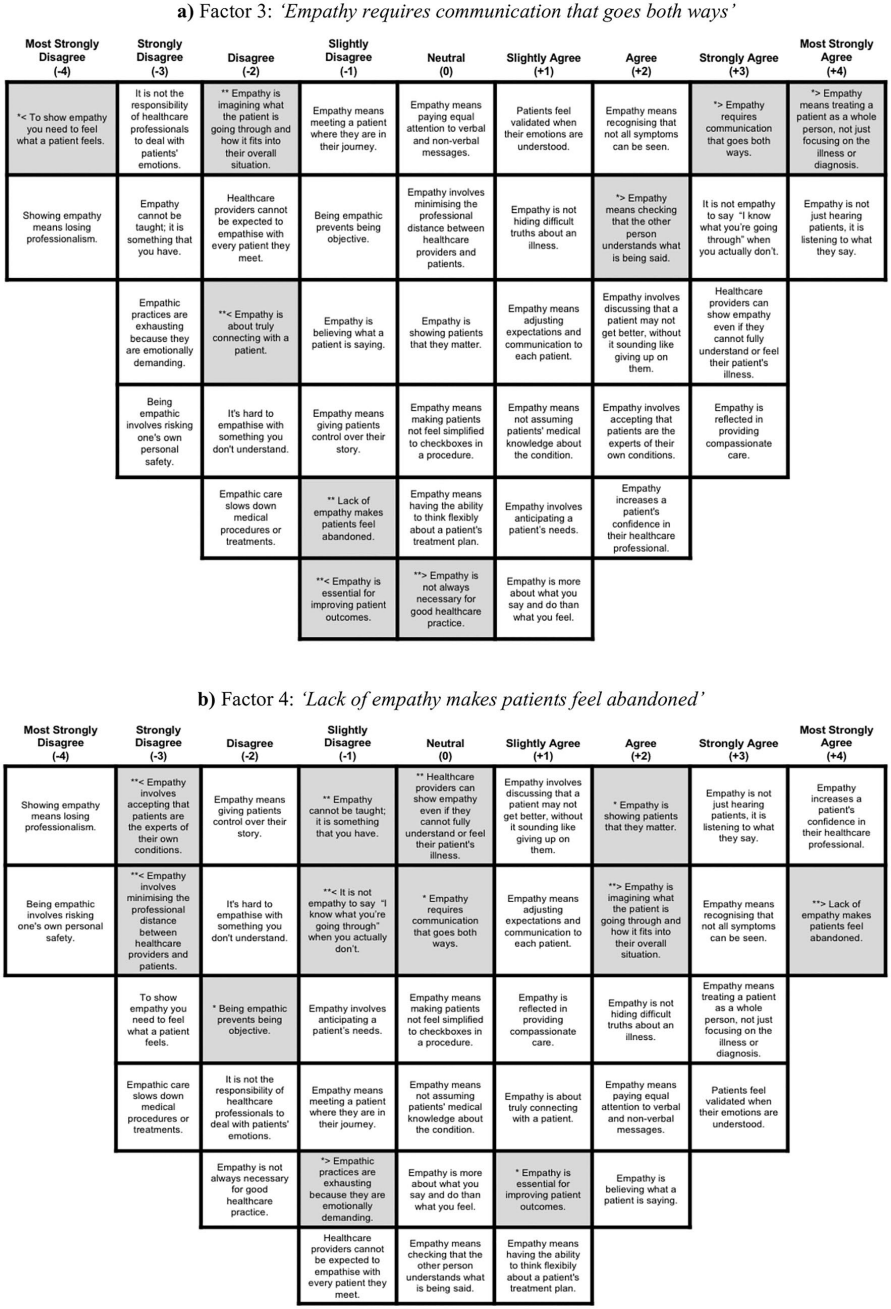
Composite Q-sorts for Factors 3 (panel a) and 4 (panel b). NOTE: Distinguishing statements are highlighted in grey. * = statistically distinguishing statement at .05 level; ** = statistically distinguishing statement at .01 level; ≥ z score indicates higher loading on this factor compared with other factors; ≤ z score indicates lower loading on this factor compared with other factors. Colour-coded versions of these grids are provided in Supplementary Figures S6 and S7

The Q-sort was implemented as an online survey on QuestionPro (https://www.questionpro.com/). To evaluate the level of agreement/disagreement with the opinion statements on clinical empathy, participants were asked to arrange the 40 items on a pre-defined Q-sort grid on a 9-point Likert scale ranging from “Most strongly disagree” (− 4) to “Most strongly agree” (+ 4) (see Figs. 2 and 3 for the grid layout). Each cell within the grid represented a single placement for one statement, resulting in a forced-choice distribution where the extreme rankings have the fewest items (Watts & Stenner, 2012). This quasi-normal distribution of data in a symmetrical pattern reveals diversity in participants’ responses, which is essential for making statistical inferences (Kamperman et al., 2020). By sorting the statements into the grid, each participant produced a completed Q-sort, generating quantitative data that captured how closely each statement aligned with their conceptualisation of empathy in healthcare interactions.

### Procedure

Participants in each group received tailored advertisements specific to their population, which included a link to the survey. Participants completed a screening questionnaire to confirm their eligibility. Demographic data were also collected, including age, gender, ethnicity, and non-identifiable information about their professional role (for HCPs) or health condition and access to medical services (for patients).

Participants were instructed to read the randomly ordered Q-set statements and sort them by clicking and dragging each item into a box. After completing the task, participants were invited to provide open-ended feedback on their experience of the sorting process and the items placed in the most extreme categories. These qualitative comments were used to enrich the interpretation of the resulting factors. Participants were also encouraged to suggest missing statements and share general reflections on the task to help contextualise findings (see Table S3).

Participants received a £10 Amazon voucher. Data were collected between January and June 2025.

### Plan of Analysis

Quantitative data were analysed following standard Q-methodological procedures using KenQ Analysis Desktop Edition (KADE; Banasick, 2023). The analysis produces automatically generated composite Q-sorts, which are hypothetical sorting patterns characterising each factor. These composite Q-sorts visually and numerically summarise how each factor prioritises the full set of statements. A by-person factor analysis with centroid extraction and varimax rotation was specified, consistent with standard practices in healthcare-focussed Q-studies (Churruca et al., 2021). In this approach, opinion statements constitute the sample, and participants serve as variables. Inter-correlations amongst participants’ Q-sorts reveal patterns of similarity, which facilitate the extraction of factors representing common perspectives.

The final factor solution was determined by inspecting the scree plot and retaining factors with Eigenvalues greater than 1. Participants were then assigned to their strongest-loading factor. Interpretation of the final factors involved reviewing statements ranked at the extremes of the grid and statistically distinguishing statements that were placed differently in one factor compared to others (*p* < .05 or* p* < .01). Additionally, z-scores were used to identify which items were ranked higher or lower in each factor relative to other factors, clarifying key differences in viewpoints.

Participants’ written comments about their Q-sort decisions and noteworthy statements were incorporated into the interpretation of each factor. Factor titles were derived from a single distinguishing statement strongly endorsed within each factor, further supported by participants’ qualitative comments, and, where relevant, we indicated which participant group was assigned most to the perspective. Demographic data were reviewed last to help characterise the profiles represented.

## Results

A four-factor solution was retained, explaining 51% of the total variance: Factor 1 accounted for 38%, Factor 2 for 5%, Factor 3 for 5%, and Factor 4 for 3% of the variance. Each factor is described below.

### Factor 1: “Empathy is About Truly Connecting”—The Dominant HCP View

Factor 1 was defined by 18 respondents (15 HCPs and 3 patients with FMS), representing the largest clinician perspective (see Table 1). In the composite Q-sort for Factor 1 (Fig. 2a), distinguishing statements ranked in agreement emphasised empathy as valuing patients, truly connecting with them, and meeting them in their personal journey, reflecting a strong emotional and relational approach to care. Free-text comments from individuals who loaded highly on Factor 1 reinforced this view:

HCP1: ‘Empathy is about connection - doesn’t mean feeling the same thing, but having the ability to perspective take.’

and

HCP2: ‘I think empathy is integral to supportive and compassionate healthcare, and I feel with a condition like Fibromyalgia with so many symptoms and such a nasty stigma by some, that it is important to show that even if you don’t know what that pain feels like, or what it is like to experience certain symptoms that you want to support them and truly hear them.’

Factor 1 also rejected potential “costs” of clinical empathy, strongly disagreeing with ‘*Being empathic prevents being objective*’ and ‘*Showing empathy means losing professionalism*’, supported by participants’ qualitative feedback:

HCP3: ‘… it is up to the clinician to provide boundaries and balance clinical care with emotional understanding... I believe being empathetic actually can improve objectivity, understanding the patient’s life and perspective can highlight their ability to engage with a treatment plan.’

Primarily, Factor 1 represents empathy as a professional skill that enhances, rather than undermines, objectivity and efficiency. Its affective dimension is emphasised, positioning emotional engagement, connection and collaboration as a cornerstone of empathic healthcare.

### Factor 2: "Empathy Cannot be Taught; It is Something That You Have"

Factor 2 was defined by four participants (1 HCP and 3 patients with FMS; see Table 1). Distinguishing statements emphasised practical, outcome-oriented aspects: avoiding simplifying patients to procedural checkboxes, thinking flexibly about treatment plans, and giving patients control over their story (Fig. 2b). Importantly, strong agreement with ‘*Empathy cannot be taught; it is something that you have*’ reflects a view of empathy as an innate trait, not a learnable skill.

FMS4: ‘Empathy is intrinsically possessed by the individual and is not imparted. Empathy exercises, whilst emotionally demanding, are not exhausting.’

Accordingly, less emphasis was placed on affective empathy, with participants disagreeing that ‘*Empathic practices are exhausting because they are emotionally demanding*’, ‘*Lack of empathy makes patients feel abandoned*’, and ‘*Empathy means treating a patient as a whole person, not just focussing on their illness or diagnosis*’, also indicating reduced importance on holistic understanding.

Unlike Factor 1, participants loading highly onto Factor 2 acknowledged potential costs of clinical empathy, agreeing more with ‘*Showing empathy means losing professionalism*’ and ‘*Being empathic involves risking one’s own personal safety*’:

HCP17: ‘Maintaining professional boundaries is essential, and empathy alone shouldn’t dictate healthcare decisions.’

Overall, Factor 2 conceptualises empathy as an intrinsic, practical skill that can be applied with minimal emotional involvement or collaborative partnership. It prioritises behavioural expressions of empathy over affective engagement and focuses on patient-centred outcomes and careful boundary management.

### Factor 3: “Empathy Requires Communication That Goes Both Ways”

Six respondents (1 HCP, 5 patients with FMS) defined Factor 3. Distinguishing items highlighted holistic care and behavioural aspects of empathy, including treating a patient as a whole person, engaging in two-way communication, and checking for mutual understanding (Fig. 3a). Qualitative comments reinforced this communication-driven, bidirectional view of empathy:

FMS10: ‘Empathy does require communication if both parties are to maximise the potential of a professional encounter not only for the better of the patient but also for the satisfaction of the health care professional who wants to know that they did make a difference.’

Participants loading on Factor 3 strongly disagreed with affective items such as ‘*To show empathy you need to feel what a patient feels*’ and ‘*Empathy is about truly connecting with a patient*’. Our experts by experience living with chronic pain noted that patients’ perceptions of clinical empathy can influence openness during appointments, given fears of being dismissed as “too emotional” and not taken seriously. This was echoed in participants’ comments:

FMS19: ‘My concerns are often dismissed as soon as the healthcare professional learns that I have fibromyalgia. Empathy means trusting a patient’s “gut feeling” and investigating further, even when the symptoms shown can be dismissed as part of an existing condition.’

Factor 3 emphasises the behavioural and cognitive aspects of clinical empathy whilst downplaying affective ones. However, unlike Factor 2, empathy is viewed less as an intrinsic trait and more as a learnable skill that can be demonstrated through actions, such as clear communication that fosters understanding. In this way, Factor 3 portrays clinical empathy as a set of observable practices that imply bidirectional involvement and result in a better patient-HCP relationship.

### Factor 4: “Lack of Empathy Makes Patients Feel Abandoned”—The Dominant Patient View

Factor 4 was defined by 12 respondents (3 HCPs, 9 patients with FMS), representing the largest patient perspective. The defining feature of Factor 4 (Fig. 3b) is emotional attunement, with distinguishing statements indicating agreement with ‘*Lack of empathy makes patients feel abandoned*’ and ‘*Empathy is showing patients that they matter*’. Respondents also emphasised the importance of personalised, holistic, and patient-focussed care, agreeing with ‘*Empathy is imagining what the patient is going through and how it fits into their overall situation*’ and ‘*Empathy is essential for improving patient outcomes*’. Qualitative comments further highlighted patients’ need to feel seen and validated, which is crucial for both emotional well-being and health outcomes:

FMS18: ‘Just because you don’t have something doesn’t mean you can’t put yourself in that person’s shoes and think about how it must feel for them… feeling like the medical professionals have just completely given up on you is genuinely crushing and hits your mental health hard.’

and

FMS16: ‘It’s important to be heard, not just nodded at and placed on a pile of other people who have the same thing.’

Factor 4 respondents strongly disagreed with statements suggesting that empathy involves accepting patients as experts and minimising professional distance. This reflects concerns about placing too much responsibility on patients, alongside a focus on maintaining professional boundaries for therapeutic outcomes:

FMS20: ‘Professionals should try to understand patients’ illness rather than assume we know everything that we go through.’

Taken together, Factor 4 reflects the value patients place on emotional validation and a holistic approach to empathic practices, which in turn supports both their emotional well-being and health outcomes. Whilst relational and collaborative aspects are less central, the emphasis is placed on empathy’s role in alleviating feelings of abandonment, invisibility, or dismissal, prioritising affective over behavioural or cognitive aspects.

### Summary of Factors

**Fig. 4:**
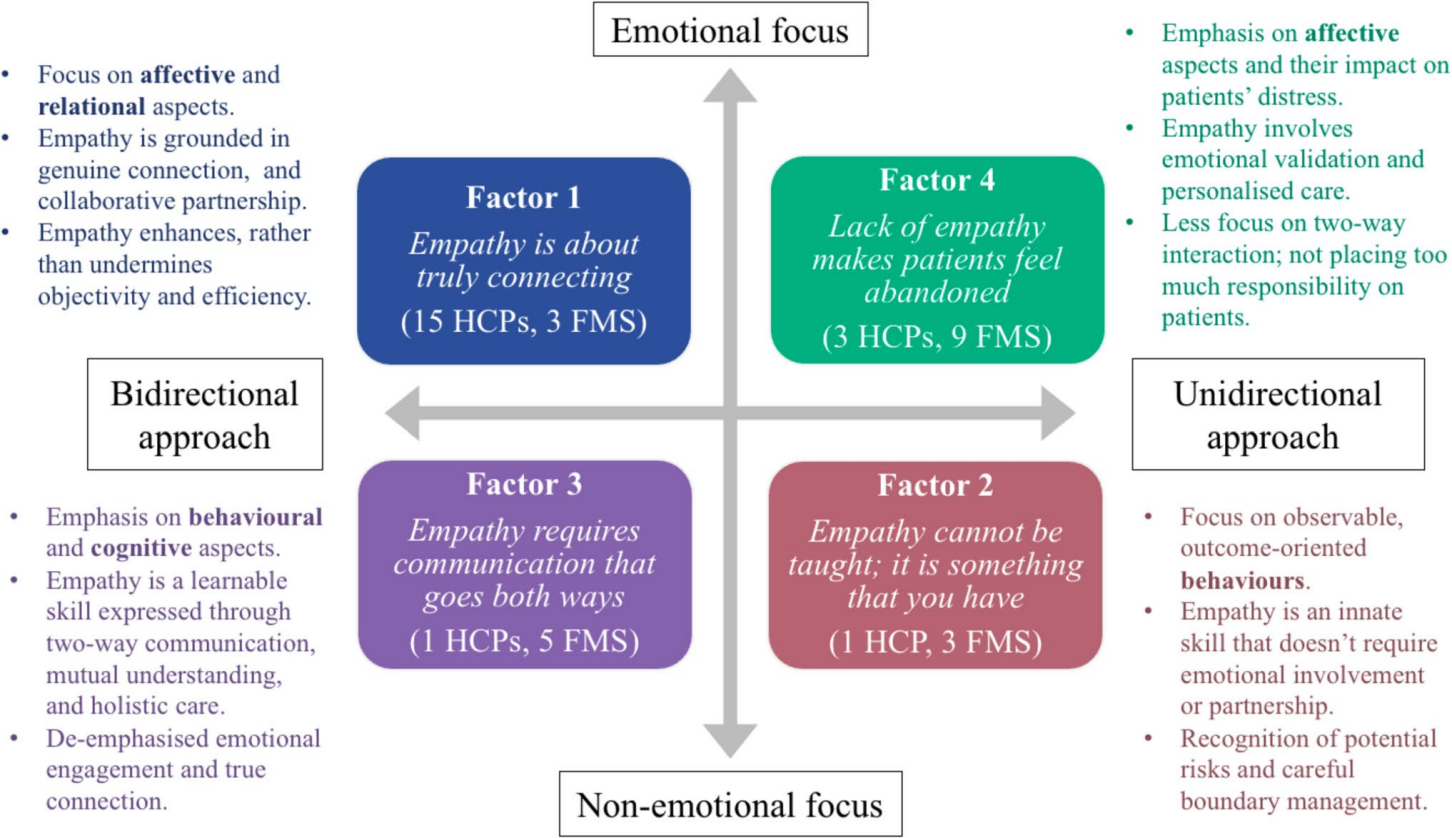
Mapping of the four factors along dimensions of affective engagement (emotional focus to non-emotional focus) and reciprocity (bidirectional to unidirectional approach)

In Fig. 4, we summarise the interplay of factors across two key dimensions identified from the data: affective engagement (i.e., emotional to non-emotional focus) and reciprocity (i.e., bidirectional to unidirectional approach). The former reflects the extent to which participants in each factor emphasised the affective component of empathy. The latter captures whether empathy was understood as a two-way relationship, involving shared engagement between patients and HCPs, or as a one-way effort only delivered by HCPs. The factors are also presented within a framework of affective, cognitive, and behavioural dimensions.^1^ In Fig. 4, the first point in each factor description indicates the empathy dimension most strongly emphasised by that viewpoint.

## Discussion

This study examined how patients with Fibromyalgia Syndrome (FMS) and healthcare professionals (HCPs) conceptualise empathy in clinical settings. Consistent with the multidimensional nature of clinical empathy (Hojat, 2016; Morse et al., 1992), four distinct factors were found, reflecting varying emphases on affective, cognitive, and behavioural components. Most HCPs clustered in Factor 1, *‘Empathy is about truly connecting’,* describing empathy as an interpersonal partnership grounded in emotional connection, whilst downplaying its potential burdens. The high representation of HCPs in mental health (*n =* 7) and advanced pain care (*n* = 12) may reflect shared professional training or vocational values shaping empathic orientation (Yu et al., 2022). In contrast, patients’ perspectives were more diverse, spanning all four factors, three of which diverged from HCP perspectives. Factor 2, *‘Empathy cannot be taught; it is something that you have’*, portrayed empathy as a practical, innate skill with potential risks for HCPs and patients, de-emphasising emotional engagement. Factor 3, ‘*Empathy requires communication that goes both ways’*, framed empathy as a learnable, action-oriented practice prioritising behavioural and cognitive dimensions. Factor 4, *‘Lack of empathy makes patients feel abandoned’,* the largest patient perspective, emphasised emotional validation and its impact on patient’s wellbeing.

We identified two higher-order dimensions from these factors: affective engagement and reciprocity. Factors 1 and 4—the predominant clinician and patient perspectives, respectively—emphasised affective empathy, consistent with evidence that emotional engagement fosters trust and strengthens relationships in pain healthcare (Murinson et al., 2011). In the context of stigmatised conditions like FMS, affective empathy may be particularly important because it underpins emotional validation, an empathic response that increases patient satisfaction and reduces many psychosocial stresses of chronic pain (Nicola et al., 2022; Vangronsveld & Linton, 2012).

However, Factors 2 and 3—endorsed by both groups but predominantly patients—prioritised behavioural and communicative facets of empathy over emotional aspects. This shared de-emphasising of affective empathy may reflect parallel self-protective strategies: patients downplay emotional expression to avoid dismissal of their suffering (Williams, 2016), whilst HCPs regulate emotion to preserve professional boundaries and mitigate compassion fatigue (Decety & Fotopoulou, 2015; Decety et al., 2010). Too much emotional involvement can distort clinical judgement and exacerbate burnout for HCPs (Stefanello, 2022), undermining care quality and reinforcing patients’ feelings of invalidation and mistrust (Kachaner et al., 2023).

These differences in affective engagement have practical implications for patient care. Repeated experiences of being disbelieved and feeling invisible, common in chronic pain in general (Hickling et al., 2024) and FMS specifically (Colombo et al., 2025) may lead some patients to prioritise observable clinician behaviours that seem more likely to secure credibility and effective care than emotion-sharing.(Graugaard et al., 2004; Maher & Gaffiero, 2025; Newton et al., 2013) These include clear and supportive communication, attentive listening, taking the patient’s concerns seriously, and becoming better educated about FMS—behaviours that patients report as key contributors to positive healthcare experiences and satisfaction (Egeli et al., 2008). Such actions can signal recognition and understanding, helping patients feel heard, respected, and supported even when emotional engagement is limited.

Beyond affective and behavioural dimensions, respondents also diverged on reciprocity. In Factors 1 and 3, empathy was conceptualised as bidirectional, involving a patient-provider partnership. This aligns with evidence that patients value active participation in care, and both patients and HCPs rank genuine partnership as a priority in chronic pain management (Slater et al., 2022). Neuroscience findings further show that mutual engagement enhances brain-to-brain synchrony, therapeutic alliance, and outcomes (Ellingsen et al., 2023). Conversely, in Factors 2 and 4—predominantly patients’ views—empathy was conceived as more unidirectional, delivered by HCPs. This may reflect the functional approach some patients with FMS adopt when navigating fragmented care, where frequent encounters with new clinicians make them prioritise outcomes over relationship-building. Such challenges can also signal frustration and mistrust from previous invalidation (Colombo et al., 2025), reinforcing a one-sided model of empathy that limits collaborative healthcare.

Our findings emphasise the salience of interpersonal dynamics in FMS care, where psychosocial factors shape clinician attitudes, patient trust, and treatment adherence (Fiske, 2024; Rowe et al., 2019). We identified differences in how clinical empathy is conceptualised, underscoring its complexity beyond emotional resonance, perspective-taking, or prosocial behaviours considered in isolation. In our sample, HCPs largely converged on an affective and relational view of empathy, whereas patients expressed a wider range of orientations across empathy dimensions, often diverging from HCPs’ perspectives. Such differences can create misalignment and cycles of miscommunication in consultations (Briones-Vozmediano et al., 2013; Byrne et al., 2023), highlighting the need for interventions that acknowledge differing conceptualisations of empathy and promote shared understanding between patients and HCPs. Although progress is limited by inconsistent definitions and measurement, specialised programmes show promise for enhancing HCPs’ empathic practices (Fuller et al., 2024; Stepien & Baernstein, 2006).

This study highlights the importance of tailoring healthcare interactions to patients’ viewpoints on empathy—particularly affective engagement and reciprocity—which may enhance clinical encounters. This is particularly important as negative healthcare experiences may reflect and reinforce FMS-related cognitive biases, making patients more likely to negatively interpret ambiguous empathy-related cues from HCPs ((Planes Alias et al., in revision). Whilst organisational support—including adequate time, supervision, and sustainable workloads—is beneficial for facilitating and sustaining empathic practices, HCPs can still take meaningful actions within systemic limitations. Positive patient experiences often result from clinicians’ individual efforts to recognise the complexities of patients’ experiences, build trust, and adopt a biopsychosocial, multidisciplinary approach (Nishikawara et al., 2023). Even brief interactions can foster a sense of connection when clinicians effectively demonstrate respect, belief in the patient, and a genuine desire to help (Nygren Zotterman et al., 2016). Small, non-time-consuming gestures—such as greeting the patient warmly, maintaining eye contact, leaning forward, and offering nonverbal affirmations—can strengthen the patient-clinician relationship and improve patient experiences, particularly for those with complex chronic pain conditions (Vorensky et al., 2024).

It is noteworthy that in our sample, demographic and clinical variables (e.g., gender, FMS duration, HCPs’ time working in healthcare) were not clearly associated with different conceptualisations of empathy. We did not capture broader contextual or psychosocial influences—such as personality traits, professional training backgrounds, or prior healthcare experiences—that may shape perceptions of empathic interactions. Future research could measure psychological constructs, such as perceived invalidation (patients) and clinician burnout (HCPs) to investigate whether these are related to alignment with certain factors, as we have proposed. Moreover, incorporating measures of perceived stigma could also clarify how interpersonal dynamics affect both patients’ and HCPs’ viewpoints, particularly in conditions that are often misunderstood or delegitimised.

Although a lack of empathy and validation is reported across other chronic pain conditions (e.g., irritable bowel syndrome, Halpert et al., 2010; endometriosis, Bullo & Weckesser, 2021), FMS may present distinct challenges that warrant focussed examination. It is often characterised by limited medical explanation, low healthcare understanding, and reduced social recognition (Album & Westin, 2008; Hellström et al., 1998), which heightens the impact of social responses to pain (Kool et al., 2009). To ensure broader relevance, the study materials were co-designed with contributors living with FMS and/or other chronic pain conditions, allowing insights to inform both FMS-specific care and future research on chronic pain more generally. Therefore, a strength of our study was the careful co-creation of the Q-set, involving experts by experience and several rounds of item refinement. We also recruited patients from a tertiary care pain registry, ensuring they met diagnostic criteria for FMS.

A limitation of the study is that HCPs were recruited through personal networks, and most were specialist pain practitioners with advanced or consultant-level expertise. This might bias findings towards perspectives of highly experienced clinicians. Moreover, because pain specialists often cultivate heightened empathy for patients experiencing pain, their responses may not reflect the broader attitudes of the general healthcare workforce. We also cannot rule out that HCPs’ responses may reflect general or aspirational models of empathy, which could differ on a patient-by-patient basis and from the practical realities in everyday clinical settings. Supporting this idea, a Q-sort study of HCPs’ attitudes towards FMS (Scott et al., 2023) found that whilst clinicians’ rankings reflected supportive attitudes, interviews revealed more transactional relationships with patients. This discrepancy suggests that HCPs’ responses may be influenced in part by social desirability or concern for professional reputation, and reinforces the need to study behaviours as well as cognitive conceptualisations of empathy. Moreover, it should be noted that Q-sort findings should not be generalised beyond the sample. Nevertheless, our study underscores that diverse conceptualisations of clinical empathy exist within and across patients with FMS and HCPs, highlighting degree of emotional engagement and reciprocity as dimensions for further exploration to ultimately enhance empathy in FMS healthcare encounters.

## Supplementary Information

Below is the link to the electronic supplementary material.

Supplementary file1 (DOCX 6852 KB)

## Data Availability

Anonymous data will be uploaded on the Open Science Framework in line with Open Science principles and the Open Research statement:[ https:/www.ljmu.ac.uk/ris/research-excellence/open-research-statement]. Data that support the findings of this study are available from the corresponding author upon request.

## Footnotes

1 To aid visualisation of the multidimensional nature of empathy, Figures S4–S7 present the composite Q-grids for each factor, with items colour-coded by affective, cognitive, and behavioural dimensions. Statements that were similarly ranked across all factors are shown in Table S4.
