# Supplementary material for: Conceptualisation of Empathy in Interactions Between Healthcare Professionals and People With Fibromyalgia Syndrome: A Mixed-Methods Study
Source: J Clin Psychol Med Settings. 2025 Dec 29;33(2):393–406. doi: 10.1007/s10880-025-10117-w (PMC13226355; doi:10.1007/s10880-025-10117-w)
Supplement: Supplementary file 1 — Supplementary file1 (DOCX 6852 KB) [file 10880_2025_10117_MOESM1_ESM.docx]

***Supplementary Materials***

**Materials and measures**

**Detailed description of the study phases**

Step 1 laid the foundation for the Q-set through a comprehensive literature review on the role of empathy in psychology and pain medicine, exploring definitions, theoretical frameworks, and tools to assess empathy in healthcare settings (Moudatsou et al., 2020). The identified key facets of empathy in chronic pain care (Kool et al., 2009; Scott et al., 2023) directly informed the design of the Q-set, alongside insights from collaborative sessions with experts by experience: people living with chronic pain and healthcare professionals (HCPs) who work extensively with patients diagnosed with FMS.

The collaborative process involved several contributors in online sessions. In Step 2, four individuals living with chronic pain (one with FMS) participated in a brainstorming session to discuss key aspects of clinical empathy. At the beginning of each consultation, the researcher provided an overview of the study's objectives, design, and main concepts. The definition of empathy, particularly in healthcare settings, was introduced, along with an explanation of its three primary components: affective, cognitive, and behavioural dimensions (Weisz & Cikara, 2021). Based on their input, an initial concourse of 100 statements was co-developed and revised with the same collaborators and two HCPs: a physiotherapist and a clinical psychologist in pain management. These professionals separately contributed to refining the Q-set (Stage 3) and piloting the Q-sort task (Stage 4). Finally, two previous and three new contributors with chronic pain piloted the survey and provided crucial feedback that helped refine the final Q-set and sorting task.

To preserve the authenticity of expert perspectives, the Q-set was developed following established guidelines for statement construction in Q methodology (Baker et al., 2017). This included retaining the original wording of participants’ expressions wherever possible, avoiding overly long or complex formulations, and ensuring that no single statement combined multiple points of view. Consistent with recommendations from prior Q-studies in health research (Akhtar-Danesh et al., 2008), we also adopted a structured, iterative process to enhance clarity, neutrality, and applicability across pain care settings. Statements were revised to remove context-specific or individual references while remaining accessible and understandable. All phases were further informed by discussions with supervisors, experts in pain and empathy research.

Stage 3 focused on refining the 100 initial statements to ensure the Q-set adequately represented the core themes of clinical empathy. Using a structured process, the concourse was systematically narrowed from 100 to 40 statements, guided by two decision trees adapted from Bell et al. (2025): one for assessing each statement individually, and another for evaluating the Q-set as a whole (see Figure S1). The first decision tree focused on assessing the relevance and clarity of each statement, ensuring it was specifically related to clinical empathy rather than broader concepts such as good clinical practice. Statements were evaluated for readability, sensitivity, and neutrality, ensuring they expressed a single, clear idea. Additionally, statements were reviewed to determine whether they could be interpreted from both the patient’s and healthcare professional’s perspectives and whether they captured a key aspect of empathy. The second decision tree aimed to eliminate redundancy by identifying duplicated or similar statements and assessing whether all statements allowed for varied interpretations. The Q-set was also examined to ensure it covered a wide range of relevant topics, represented diverse facets of empathy in healthcare interactions, and was generalisable across different healthcare contexts, including other chronic pain conditions. Statements that did not meet these criteria were revised or removed (see Figure S2).


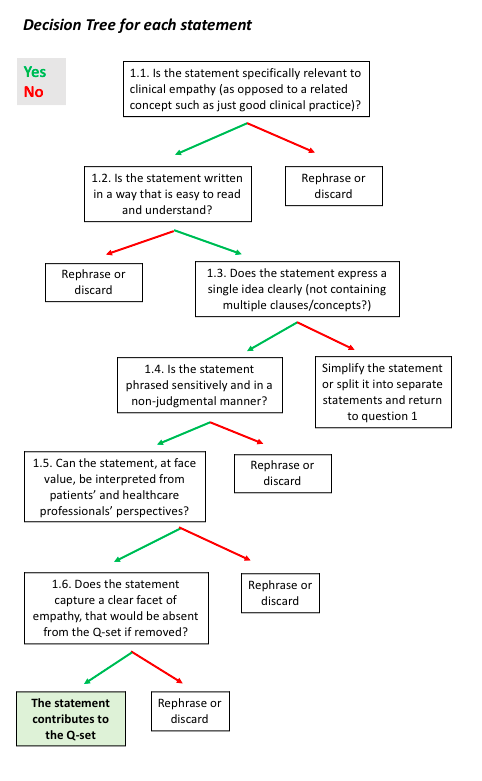

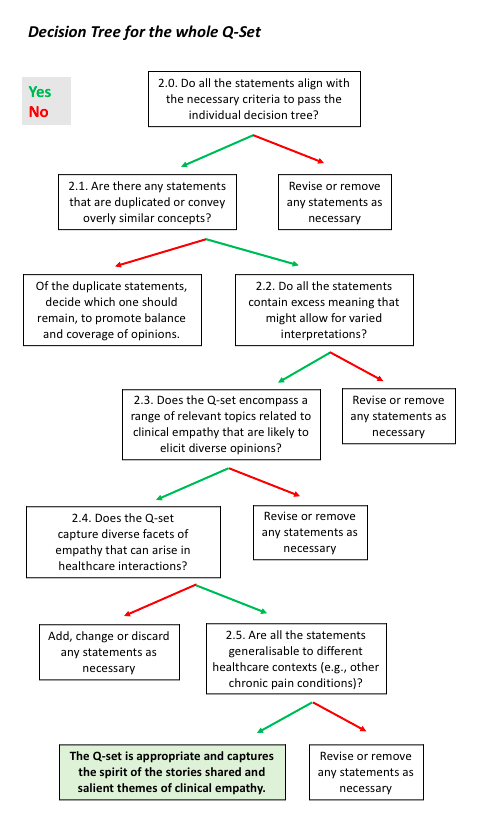


**Figure S1.** *Q-set decision trees*

NOTE. Diagram 1.a) illustrates the Decision Tree applied to evaluate each individual statement, while Diagram 1.b) depicts the Decision Tree used for refining the entire Q-set


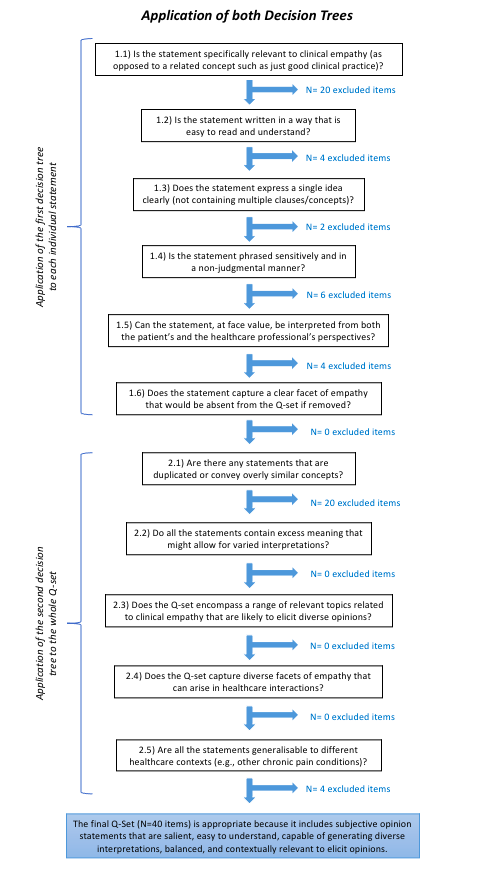
**Figure S2.** *Q-set refinement process using both decision trees.*

NOTE. *N* = number of statements removed for not meeting the inclusion criteria.**Results**

**Colour-coded composite Q-sort grids by factor**

The composite Q-grids resulting from the factor analysis were colour-coded to reflect the multidimensional nature of empathy, enhancing clarity of interpretation. The three dissociable yet interrelated components — affective, cognitive, and behavioural — have been widely recognised in both psychological models of empathy (Weisz & Cikara, 2021) and theoretical frameworks of clinical empathy (Decety & Jackson, 2004; Morse et al., 1992).

**
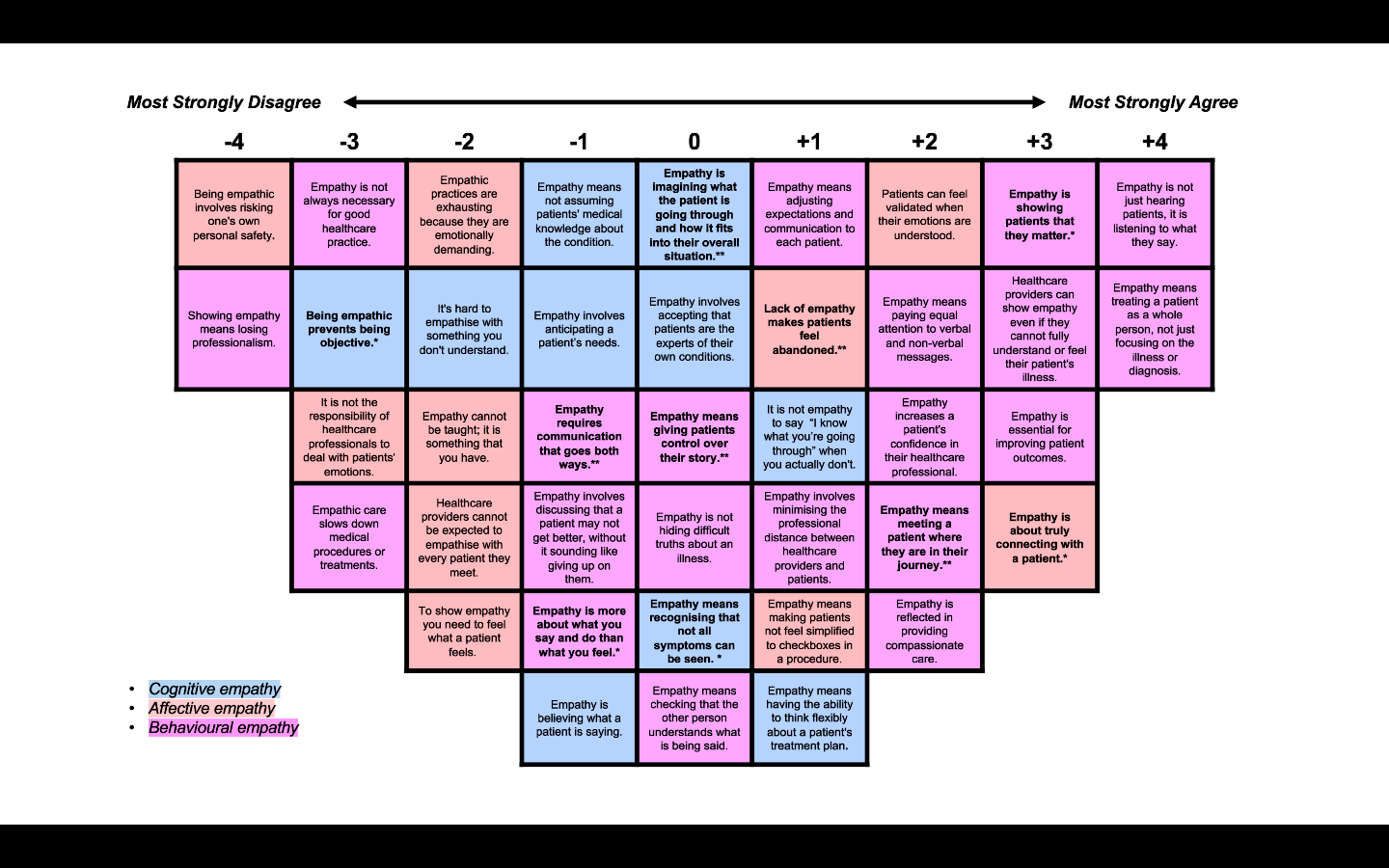
Figure S4.** *Composite Q-sorts for Factor 1 ‘Empathy is about truly connecting — the dominant healthcare professional view*

NOTE. Blue boxes represent statements reflecting the cognitive dimension of empathy, orange boxes indicate the affective dimension, and purple boxes correspond to the behavioural dimension. Distinguishing statements: * = statistically distinguishing statement at .05 level; ** = statistically distinguishing statement at .01 level.


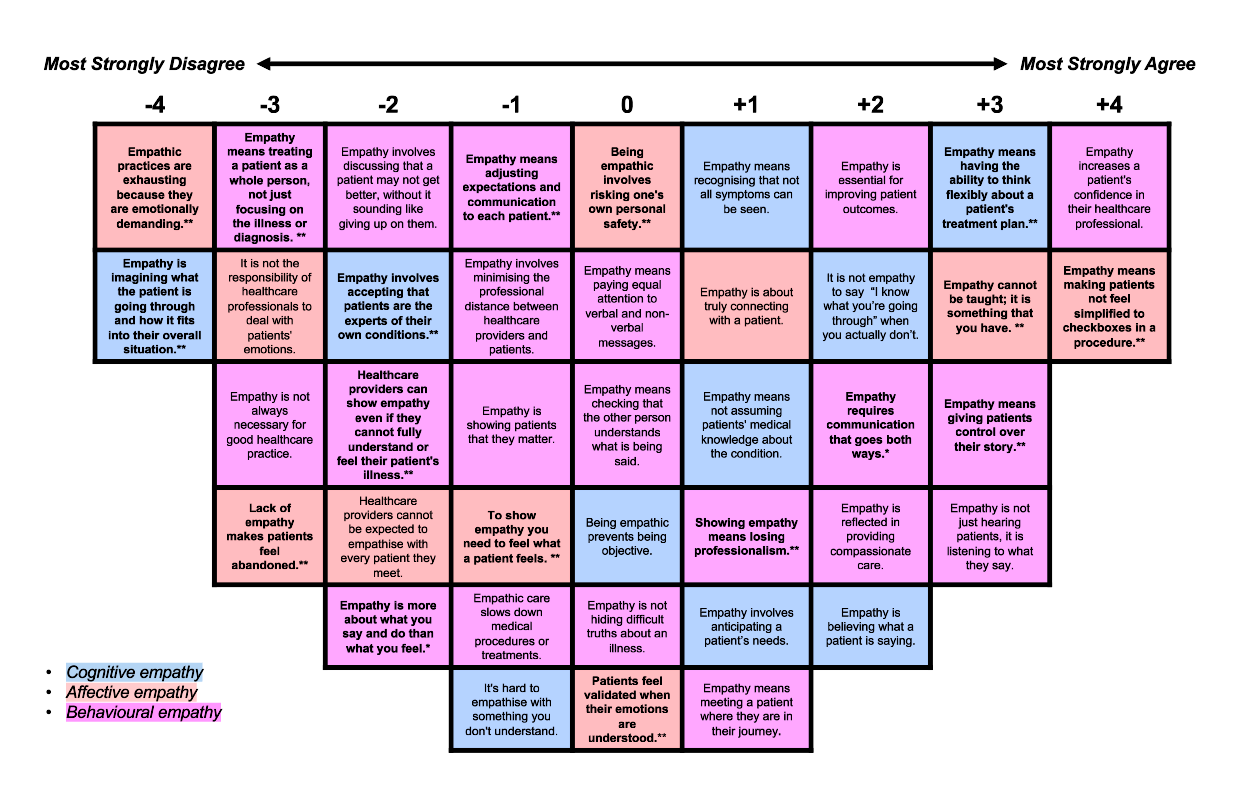
**Figure S5.** *Composite Q-sort for Factor 2 ‘Empathy cannot be taught; it is something that you have’*

NOTE. Blue = cognitive empathy, orange = affective empathy, purple = behavioural empathy. Distinguishing statements: * = statistically distinguishing statement at .05 level; ** = statistically distinguishing statement at .01 level.


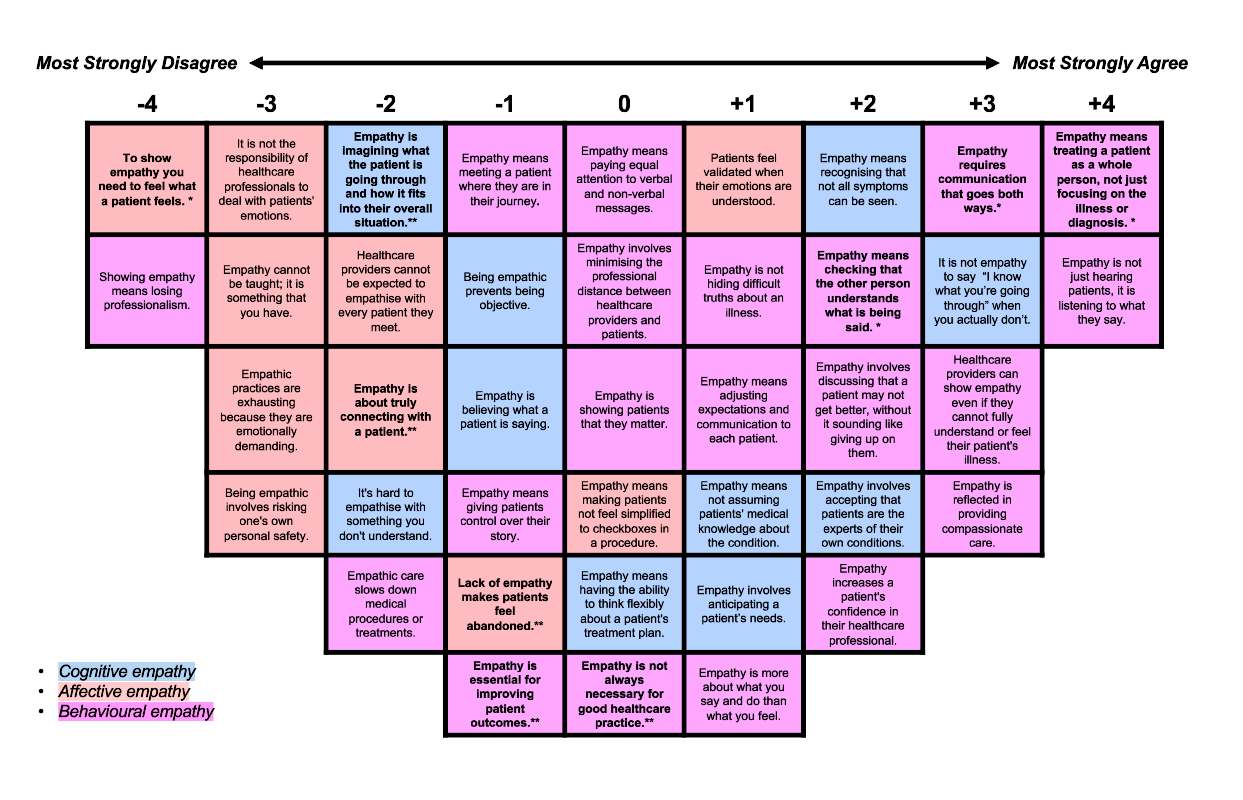
**Figure S6.** *Composite Q-sort for Factor 3 ‘Empathy requires communication that goes both ways’*

NOTE. Blue = cognitive empathy, orange = affective empathy, purple = behavioural empathy. Distinguishing statements: * = statistically distinguishing statement at .05 level; ** = statistically distinguishing statement at .01 level.

**
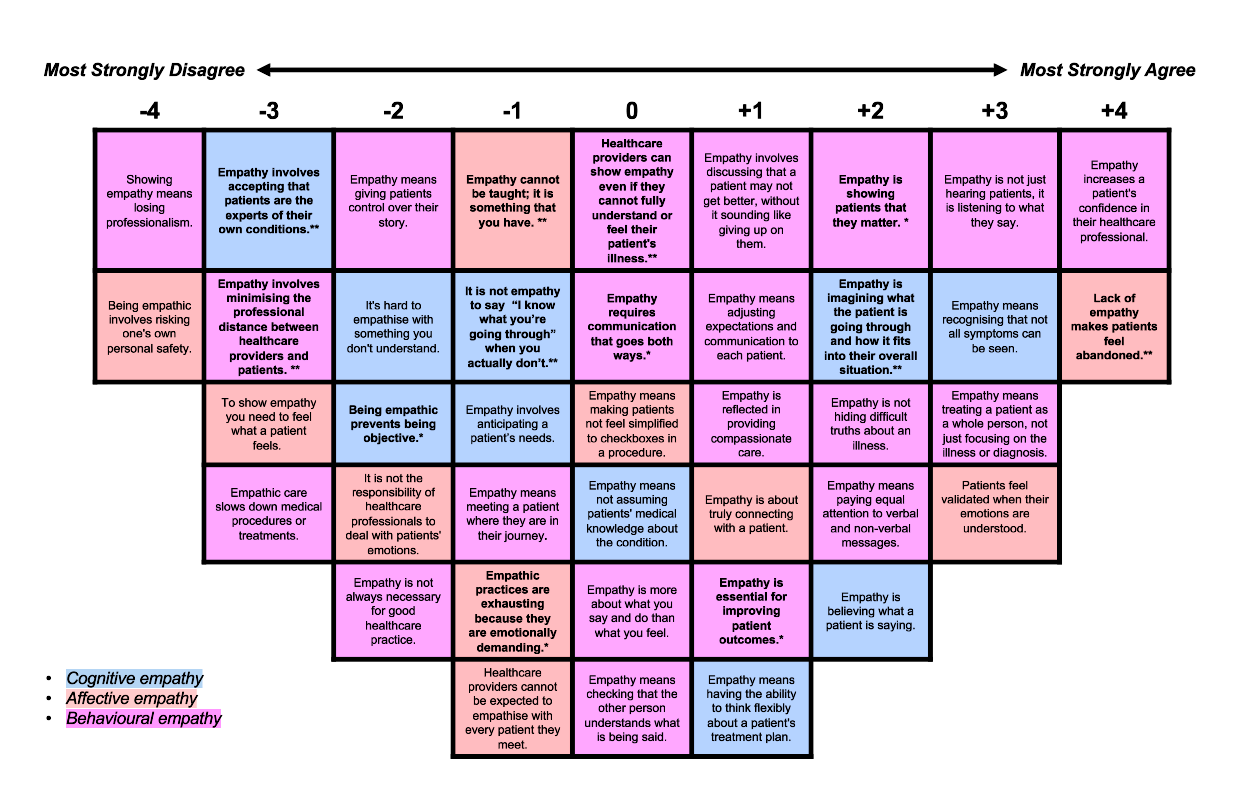
Figure S7.** *Composite Q-sort for Factor 4 ‘Lack of empathy makes patients feel abandoned — the dominant patient view’*

NOTE. Blue = cognitive empathy, orange = affective empathy, purple = behavioural empathy. Distinguishing statements: * = statistically distinguishing statement at .05 level; ** = statistically distinguishing statement at .01 level.

**Open-ended questions to ask for qualitative feedback**

**Table S3.** *Qualitative questions and sample participants’ responses.*

| Questions | Relevant answers |
| --- | --- |
| 1. Please share your overall experience with the sorting process. | - *It was hard to place statement in the middle sections. I could identify clear extremes very easily, those statements that I clearly disagreed or 100% agreed with.* – HCP10, Factor 1 - *Difficult to put them in all of the boxes. Sometimes I thought that one statement could be in more than one box. It is difficult sometimes to separate empathy and compassion - compassion allows you to respond and listen to a patient in a supportive way without the emotional demand especially when staff may near burnout*. – HCP16, Factor 4 - *It was quite thought provoking. At different times in my journey so far, I may have answered differently. Very hard to have specific numbers in each category.* – FMS10, Factor 3 - *A little tricky only being able to out a certain amount in each box but understandable, you have to think more about what you truly perceive to be more important.* – FMS18, Factor 4 |
| 1. Were there any specific statements that stood out to you as particularly significant or noteworthy? | - *The idea that empathy means being less professional - I think some medical professionals struggle to see that empathy doesn't make them soft or mean that they have to agree with the patient*. – HCP15, Factor 1 - *The statement about meeting a patient on their journey again is something that resonated with me. The journey with chronic pain is different for everyone and a patient will feel different on two separate occasions.* – HCP20, Factor 1 - *The statement ‘Doctors should validate a patient’s experience even if they don’t fully understand it’ really hit home. As a fibromyalgia patient, having my struggles recognised means everything, even if docs can’t wrap their heads around it all.* – FMS3, Factor 1 - *“Empathy can't be taught, it's something you have” I think this one fits the bill*. – FMS4, Factor 2 |
| 1. Why did you place these two statements in the "Most Strongly Disagree" (-4) category? | - *I think empathy can be taught - but that not every HCP is teach-able.* – HCP6, Factor 1 - *You can't get too 'bogged down' with the patient’s challenges. As the professional, you are the one who is working with the patient to help them 'climb out of the hole', not get stuck down there with them. You need to be okay yourself to help others.* – HCP13, Factor 1 - *Because I feel these are being led by patients’ thoughts of overcontrolling situations rather than what’s best.* – FMS7, Factor 4 - *Being a healthcare professional and knowing the medical reasons behind a patient’s feelings/pain/etc should negate the need for having felt that condition for themselves to empathise with the patient.* – FMS19, Factor 3 |
| 1. Why did you place these two statements in the "Most Strongly Agree" (+4) category? | - *It's not enough to hear, we have to truly listen to what patients are saying. I think empathy hugely improves outcomes. When people feel heard they can more readily drop the fight to show people how hard things are, and getting better can feel less threatening.* – HCP5, Factor 1 - *The recognition that a lack of empathy can lead to patients feeling not listened too/or abandoned - see this more and more often. Links in also to not feeling believed or getting enough time to share the information they think is important.* – HCP16, Factor 4 - *As a fibromyalgia patient, having my pain and struggles acknowledged means the world. Even if docs can’t grasp every detail, just saying ‘I see you’ is huge.* – FMS3, Factor 1 - *It is not just good enough to say you understand when truly as a patient you get to know when the provider is just paying lip service.* – FMS9, Factor 4 |
| 1. If you feel any important statements were missing from this task, please suggest them. | - *Something about difference between empathy and sympathy.* – HCP1, Factor 1 - *Empathy is about providing a space to share without fear of judgement or disbelief.* – HCP12, Factor 4 - *Empathy means acknowledging the patient's pain as real, even when it cannot be fully measured or medically explained.* – FMS1, Factor 3 - *Lack of knowledge about fibromyalgia among healthcare professionals can hinder their ability to express empathy.* – FMS2, Factor 2 - *Empathy and compassion go hand in hand but with a chronic pain condition it is difficult not to judge so possibly “empathy can be judgmental”* – FMS9, Factor 4 |

**Consensus statements**

**Table S4.** *Q-sort statements similarly ranked across all factors.*

| Ranking Category | Statement |
| --- | --- |
| Disagreement (columns –4, -3, -2) | - *It is not the responsibility of healthcare professionals to deal with patients' emotions (S3)* |
| Neutral  (columns –1, 0, +1) | - *Empathy means not assuming patients' medical knowledge about their condition (S14)* - *Empathy involves anticipating a patient’s needs (S17)* - *Empathy means adjusting expectations and communication to each patient (S23)* |
| Agreement  (columns +2, +3, +4) | - *Empathy increases a patient's confidence in their healthcare professional (S1)* - *Empathy is not just hearing patients, it is listening to what they say (S27)* |

NOTE. The ranking categories correspond to the degree of consensus, with “Disagreement” indicating strong rejection of the statement, “Neutral” reflecting ambivalence or context-dependence, and “Agreement” indicating strong endorsement. Lowest ranked statement: S3 (-3, -3, -3, -2). Neutral ranked statements: S14 (-1, +1, +1, 0), S17 (-1, +1, +1, -1), and S23 (+1, -1, +1, +1). Highest ranked statements: S1 (+2, +4, +2, +4), and S27 (+4, +3, +4, +3).

**References**

Akhtar-Danesh, N., Baumann, A., & Cordingley, L. (2008). Q-Methodology in Nursing Research: A Promising Method for the Study of Subjectivity. *Western Journal of Nursing Research*, *30*(6), 759–773. https://doi.org/10.1177/0193945907312979

Baker, R., McHugh, N. A., & Mason, H. (2017). Constructing statements for use in Q methodology studies. In J. Coast (Ed.), *Qualitative Methods for Health Economics.* (pp. 163–174). Rowman & Littlefield International Ltd. https://rowman.com/ISBN/9781783485628/Qualitative-Methods-for-Health-Economics#

Bell, V., Murray, E., Muñoz, L., & Krahé, C. (2025). In harm’s way: Moral injury and the erosion of trust for emergency responders in the United Kingdom. *European Journal of Psychotraumatology*, *16*(1), 2513107. https://doi.org/10.1080/20008066.2025.2513107

Decety, J., & Jackson, P. L. (2004). The Functional Architecture of Human Empathy. *Behavioral and Cognitive Neuroscience Reviews*, *3*(2), 71–100. https://doi.org/10.1177/1534582304267187

Kool, M. B., van Middendorp, H., Boeije, H. R., & Geenen, R. (2009). Understanding the lack of understanding: Invalidation from the perspective of the patient with fibromyalgia. *Arthritis Care & Research*, *61*(12), 1650–1656. https://doi.org/10.1002/art.24922

Morse, J. M., Anderson, G., Bottorff, J. L., Yonge, O., O’Brien, B., Solberg, S. M., & McIlveen, K. H. (1992). Exploring Empathy: A Conceptual Fit for Nursing Practice? *Image: The Journal of Nursing Scholarship*, *24*(4), 273–280. https://doi.org/10.1111/j.1547-5069.1992.tb00733.x

Moudatsou, M., Stavropoulou, A., Philalithis, A., & Koukouli, S. (2020). The Role of Empathy in Health and Social Care Professionals. *Healthcare*, *8*(1), 26. https://doi.org/10.3390/healthcare8010026

Scott, L., Dolan, E., Baker, N., & Melia, Y. (2023). Exploring attitudes of healthcare professionals towards those with fibromyalgia: A Q-methodological approach. *British Journal of Pain*, *17*(4), 352–365. https://doi.org/10.1177/20494637231159502

Weisz, E., & Cikara, M. (2021). Strategic Regulation of Empathy. *Trends in Cognitive Sciences*, *25*(3), 213–227. https://doi.org/10.1016/j.tics.2020.12.002
